# Chemical Composition, Starch Digestibility and Antioxidant Capacity of Tortilla Made with a Blend of Quality Protein Maize and Black Bean

**DOI:** 10.3390/ijms13010286

**Published:** 2011-12-27

**Authors:** Eva M. Grajales-García, Perla Osorio-Díaz, Isabel Goñi, Deisy Hervert-Hernández, Salvador H. Guzmán-Maldonado, Luis A. Bello-Pérez

**Affiliations:** 1 Centro de Desarrollo de Productos Bióticos del IPN, Km 8.5 carr, Yautepec-Jojutla, Colonia San Isidro, Apartado postal 24, Yautepec, Morelos 62731, Mexico; E-Mails: evamariagg05@hotmail.com (E.M.G.-G.); labellop@ipn.mx (L.A.B.-P.); 2 Departamento de Nutrición, Facultad de Farmacia, Universidad Complutense de Madrid, Ciudad Universitaria, Madrid 28040, Spain; E-Mails: igonic@farm.ucm.es (I.G.); deisy_hervert@hotmail.com (D.H.-H.); 3 Unidad de Biotecnología del Campo Experimental Bajío (INIFAP), Km 6.5 Celaya, San Miguel de Allende S/N, Celaya, Guanajuato 38110, Mexico; E-Mail: guzman.horacio@inifap.gob.mx

**Keywords:** tortilla, bean, starch digestibility, antioxidant capacity, chemical composition

## Abstract

Tortilla and beans are the basic components in the diet of people in the urban and rural areas of Mexico. Quality protein maize is suggested for tortilla preparation because it presents an increase in lysine and tryptophan levels. Beans contain important amounts of dietary fiber. The objective of this study was to prepare tortilla with bean and assesses the chemical composition, starch digestibility and antioxidant capacity using a quality protein maize variety. Tortilla with bean had higher protein, ash, dietary fiber and resistant starch content, and lower digestible starch than control tortilla. The hydrolysis rate (60 to 50%) and the predicted glycemic index (88 to 80) of tortilla decreased with the addition of bean in the blend. Extractable polyphenols and proanthocyanidins were higher in the tortilla with bean than control tortilla. This pattern produced higher antioxidant capacity of tortilla with bean (17.6 μmol Trolox eq/g) than control tortilla (7.8 μmol Trolox eq/g). The addition of bean to tortilla modified the starch digestibility and antioxidant characteristics of tortilla, obtaining a product with nutraceutical characteristics.

## 1. Introduction

Maize (*Zea mays* L.) is used in the production of tortilla, which is the principal staple food in the Mexican diet [1,2]. In the rural areas of Mexico, tortilla *per capita* consumption is higher than 120 kg/year, which is equivalent to 328 g/day [3]. It is known that maize is deficient in lysine and tryptophan, two essential amino acids. Quality protein maize (QPM) can be an alternative to improve the nutritional quality of tortilla, which was developed from opaque2 maize. QPM shows higher lysine (3.4–6.0 g/100 g of protein) and tryptophan (0.8–1.2 g/100 g of protein) content than regular maize [4].

The common bean (*Phaseolus* v*ulgaris* L.) has an important place among the legumes of major production and consumption in Africa, India, Latin America, and Mexico [5–7]. In the rural areas of Mexico, consumption of beans represents 15% of a normal diet [8]. In consequence, common bean and maize represent the main food source for more than 25 million Mexican people who live in rural areas, as well as for 30 million people who live in marginal urban areas [9]. Beans are a rich and inexpensive source of proteins (20–25 g/100 g) and carbohydrates (50–60 g/100 g) [10] and they are beneficial for health, with a low glycemic index [11]. Recently, our group reported the antioxidant capacity of three legumes consumed in Mexico. It was found that black bean had the greatest concentration of proanthocyanidins (an outstanding antioxidant) [12].

Traditionally, people in the rural areas of Mexico and Central America consume a mixture of tortilla, beans, and chili, often named “taco” [13]. It is well-known that such a mixture improves some of the nutritional characteristics of the individual items, especially on the nutritionally relevant features of the polysaccharides present in this composite food [13]. Results from our group suggested that most of the beneficial “slow release carbohydrate” features of black beans are retained by the mixed bean-tortilla meal, an observation that may provide basis for new dietary uses of these traditional foods [14].

However, the blend “masa” and cooked bean flour, for thereafter-made tortilla has not been studied in its starch digestibility and antioxidant capacity. Mora-Avilés *et al.* [15] prepared tortilla with the blend QPM and bean, and evaluated the amino acids and mineral changes that occur during nixtamalization and the chemical and nutritional characteristics of regular, commercial and QPM-bean tortilla.

Therefore, the objective of the present work was to assess the chemical composition, digestibility of starch and antioxidant capacity in tortilla prepared with the blend QPM-black bean compared to that of individual ingredients.

## 2. Results and Discussion

### 2.1. Chemical Composition

Chemical composition of raw materials and tortillas is shown in Table 1. When QPM is nixtmalized to produce “masa” and tortilla, the protein does not change appreciably. An increase of 37% in protein content in QPM-black bean tortilla was found compared to that of QPM tortilla. Our results for the protein content of QPM-black bean tortilla were higher compared to those previously reported by Hernández-Salazar *et al.* [16], who indicated that a tortilla prepared with maize-bean showed 10.5 g/100 g; however, they did not declare the maize-bean blend used. Black dry bean characterized by high protein content, shows between 18.9 and 24.2 g/100 g [17]. The presence of black bean in tortilla reported in this work was responsible for increments in protein. On the other hand, maize shows lower protein level than beans. Diverse hybrids and varieties of maize harvested in México had protein content between 8.3 and 11.3 g/100 g, with higher amount in dent and semident type grains than in crystalline and semicrystalline grains [18]. When maize is nixtamalized to produce “masa” (8.7 g/100 g) and tortilla (7.5 g/100 g), the protein does not change appreciably compare with raw maize [19]. Maize shows higher fat (6.6 g/100 g) content [19] than dry beans (1.3 and 2.8 g/100 g) [17] due to germ of the former (Table 1). Meanwhile, “masa” showed 3.1 g/100 g and tortilla of 2.5 g/100 g of fat content due to that during nixtamalization fat was eliminated in the “nejayote” [19]. Fat content in “masa” an tortilla reported previously was lower to those reported here [19]. The maize utilized here can play an important role in our results; hybrids and varieties of maize [18] had fat content between 4 and 7 g/100 g, and commercial white tortilla had a fat content of 3 g/100 g [16]. Initially, the expression of the opaque2 gene increased the content of essential amino acids in maize but was also associated with lower per-acre yields, increased susceptibility to pests and diseases, and a soft endosperm, which made it unacceptable to many potential users. Currently, QPM which is high-yielding, disease- and parasite-resistant, and has a brittle endosperm but retains the superior amino acid balance of opaque2, was achieved through the introduction of gene modifiers. These characteristics can be bred into traditional, locally adapted varieties anywhere that maize is grown [20], so, as well as in common maize varieties, the differences in fat content of QPM varieties can be depending on the variety [18,20], and in tortillas, the differences in fat content may be also attributable to maize variety and conditions prevailing during nixtamalization process [21]. QPM-black bean tortilla reported here had similar fat content than tortilla, this pattern is due to that in the blend the amount of maize is higher and no dilution effect was found.

Ash content in black bean was higher than in “masa” and tortillas (Table 1). In general, legumes are characterized by high minerals levels, which depend on the species, cultivar, and agronomic characteristics (soil type). Black bean [22] had an ash content of 5.4 g/100 g and different varieties of black bean harvested in Mexico ranged between 3.6 to 5.2 g/100 g [17]. Maize has low ash content as was reported in diverse hybrids and cultivars (1.1–1.7 g/100 g) [18]. Tortilla prepared in our laboratory shows an ash content of 1.6 g/100 g [19], meanwhile commercial white tortilla showed 1.9 g/100 g [16]. However, the QPM-black bean tortilla increased its ash contents (2.58 g/100 g) due to the contribution of the bean. Similar pattern was reported in tortilla prepared with white maize flour (1.88 g/100 g) and maize-bean flour (2.95 g/100 g) [16].

Total dietary fiber (TDF) content was higher in QPM-black bean tortilla compared to that of QPM masa and QPM tortilla (Table 1). Black bean showed the highest TDF level. Beans are characterized by a high content of this component. In two different beans (Navy and Red) the TDF contents were 36.2 and 36.8 g/100 g, respectively [23]. Reynoso-Camacho *et al.* [24] reported that the addition of common bean in the diets reduced colon cancer in Sprage-Dawley rats, and this pattern was influenced by the dietary fiber content. There were no differences between content of TDF of QPM “masa” and QPM tortilla; meanwhile, QPM-black bean tortilla showed higher value than QPM tortilla. The addition of bean in the blend increases this nutritional component in tortilla in 57%, an important issue due to that black bean tortilla could be considered as a functional food.

### 2.2. Starch Digestibility

Black bean showed the lowest, and “masa” and tortilla the highest total starch (TS) levels (Table 2). Black bean varieties harvested in Mexico shows TS between 33.6 and 36.7 g/100 g [17]; in another study, cooked black bean showed a TS content of 53.8 g/100 g [22], which was higher than those assessed here, the bean variety can be responsible of this difference. On the other hand, total starch level in masa and tortilla was similar. It has been reported that tortillas elaborated with commercial dry masa flour shows TS content between 76.2 and 79.0 g/100 g [25], and those elaborated with commercial masa between 74.8 and 79.7 g/100 g [21], values similar to those reported in this study. Black bean tortilla had lower TS content than tortilla, this effect is due to dilution produced by addition of black bean with lower TS content. Similar TS content (65.6 g/100 g) was determined in commercial maize-bean tortilla [16].

TS content is in tune with digestible starch (DS) amount recorded in the samples analyzed (Table 2). Low DS content, as was assessed in different cooked dry bean varieties harvested in Mexico, with values ranged between 21.7 and 32.2 g/100 g [26]. However, it has been reported a higher range of DS values in cooked beans (27.88–39.21 g/100 g) [27], suggesting that the cooking method, the storage and perhaps the dry bean variety play an important role in DS content, a value within this range was determined here. QPM “masa” shows the highest DS content, decreasing in QPM tortilla (Table 2). This decrease could be due to the fact that the starch of masa was partially gelatinized and part of this was retrograded when the tortilla was cooling down. The formation of retrograded starch requires dehydration of the gelatinized sample [28,29], a phenomenon that is likely to take place when tortillas are baked at ≈ 250 °C and cooled. Rendon-Villalobos *et al.* [19] reported a similar pattern, with higher DS value in “masa” (79.6 g/100 g) than tortilla (72.9 g/100 g). In addition, a similar value of DS for tortilla made with commercial “masas” was reported (70.1–76.0 g/100 g) [21], and those made with commercial dry masa flour (70.6–74.9 g/100 g) [25]. Tortilla prepared with commercial white maize flour (63.5 g/100 g) [16] and commercial tortilla (65.2 g/100 g) [13] had lower DS content. The addition of bean to tortilla decreased DS content in approximately 15.6%, this pattern due to the amount of bean added and the lower DS level in this legume. Mexican “taco” [13] (a mixture of tortilla and bean 60:40) was studied in its DS content, showing a DS content of 52.6 g/100 g; the ratio maiz:bean and the method utilized for the preparation of the samples could explain such differences. In commercial maize-bean tortilla DS content of 60.3 g/100 g was reported, but the ratio maize-bean is not declared [16].

Black bean showed the highest resistant starch (RS) content and was similar to that of QPM-black bean tortilla. RS contents between 3.5 and 5.1 g/100 g were reported in five Mexican varieties of black bean [26], other common bean varieties shows RS level of 5.4 g/100 g (Peruano) [30], 5.3 g/100 g (Cotaxtla) [13] and 0.64 g/100 g (Mayocoba) [31]. QPM masa showed the lowest RS content, and an increase was obtained in tortilla (Table 2). Rendon-Villalobos *et al.* [19] reported similar pattern, with lower RS value in masa (2.05 g/100 g) than tortilla (3.12 g/100 g). The RS content of the QPM tortilla is higher than those reported in tortilla made with commercial dry masa flour (1.20–2.46 g/100 g) [25], tortilla made with commercial masas (1.36–3.05 g/100 g) [21], commercial tortilla (2.14 g/100 g) [13] and tortilla made with commercial white maize flour (2.55 g/100 g) [16]. The QPM variety could be responsible of this high RS content in tortilla, because until today there are not reports dealing with RS content in this kind of tortilla. The addition of bean to maize produced a small increase in RS amount in tortilla because the value of QPM tortilla was high. Sáyago-Ayerdi *et al.* [13] reported in a Mexican “taco” (a mixture of tortilla and bean 60:40) a RS content of 3.93 g/100 g, and Hernández-Salazar *et al.* [16] in a commercial maize-bean tortilla a RS amount of 2.99 g/100 g. QPM-black bean tortilla has this characteristic that is important in health because recently has been considered the RS a functional ingredient to battle obesity [32].

### 2.3. *In Vitro* Kinetic of Starch Digestion

**Figure 1:**
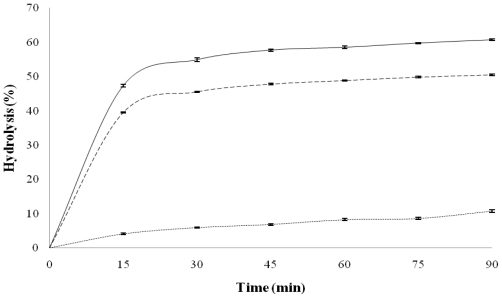
Average *in vitro* starch hydrolysis curves of black bean flour (··), quality protein maize (QPM) tortilla (—) and QPM-black bean tortilla (– –). Bars represent SEM.

Figure 1 shows *in vitro* starch hydrolysis of QPM-black bean and QPM tortillas. QPM tortilla exhibited the highest hydrolysis percentage during the assay; its amylolysis level increased quickly (inside the first 15 min), and thereafter the increase was slowest reaching approximately 55% after 30 min and did not increase at longer reaction times. Tortillas made with diverse commercial dry “masa” flours had similar pattern, but their levels of hydrolysis by the end of the assay had between 70–80% [25], and for tortillas made with diverse commercial “masas” the hydrolysis percentage were between 70–75% after 30 min of the assay and did not increase thereafter [21]. The lowest hydrolysis percentage in QPM tortilla can be due to that introduction of QPM character in maize modifies some structural characteristics of starch. A similar pattern to that of tortilla in the hydrolysis assay was found for QPM-black bean tortilla, but the hydrolysis percentage were lower, reaching at approximately 45% of hydrolysis after 30 min. The addition of the bean to maize decrease the hydrolysis rate of starch present in the blend due to that bean had the lowest hydrolysis values, reaching to hydrolysis percentage at 90 min of approximately 11%. Vargas-Torres *et al.* [26] reported hydrolysis percentage (90 min) for diverse cooked black bean varieties harvested in Mexico between 17 and 28%. Tovar *et al.* [33] reported that several factors are involved in the reduced bioavailability of legume starches. The presence of intact tissue/cell structures enclosing starch granules hinders the swelling and solubilization of starch resulting in reduced *in vitro* digestion rate.

Predicted glycemic indices (pGI) were calculated from the 90 min degree of hydrolysis values of the samples (Table 3) [34]. QPM tortilla had the highest pGI compared to the rest of samples analyzed; however, this value is lower than those determined different tortillas, where the values ranged between 102 and 108 [35], and commercial tortilla was 97.5 [36]. QPM tortilla has been shown starch digestibility parameters lower than those determined in tortillas made with commercial dry masa flours, commercial masas and commercial tortillas, the use of QPM can be important to produce tortilla with low caloric response. Cooked black bean had the lowest pGI (47.9), but using other method (chewing/dialysis test) cooked black bean had lower pGI (27) [13], and using canned black bean with chewing/dialysis method the pGI value was 44 [14]. The addition of bean decreased the pGI value of tortilla (79.8) that was higher than those determined by the chewing/dialysis method in Mexican “taco” (a mixture of tortilla and bean 60:40), using different tortilla and bean, with value of 48 (tortilla and canned black bean) [14] and 51 (tortilla and cooked black bean) [13].

### 2.4. Polyphenols Content

Polyphenols content of raw materials and tortillas is shown in Table 4. Black bean presented the highest extractable polyphenols (EP) level. Two black bean varieties showed a phenolic content of 3.37 and 6.99 mg/g, differences that can be attributed to the bean variety [37], a value within this range was determined here. EP value in tortilla was similar to those reported for yellow maize [38] with a free phenol content of 1.04 mg/g and cereals with a EP content of 1.07 mg/g [39]. The addition of bean to the blend increased the EP content in the tortilla in 107%.

Non extractable polyphenols (proanthocyanidins and hydrolysable polyphenols) in all samples showed higher value than extractable polyphenols, which is consistent with the results reported by Saura-Calixto *et al.* [39] for legumes and cereals.

It has been reported a wide range of proanthocyanidins (PA) content values in different bean varieties (16.8–38.1 mg/g [40]; 6.9–32.4 mg/g [41], the differences of PA content in beans depend on the variety and the locality [40], a value within these ranges was determined here. The PA in tortilla was not detected, as was reported by Pérez-Jiménez and Saura-Calixto [42] and Saura-Calixto *et al.* [39] for cereals. The PA was detected in tortilla added with bean; this behaviour indicates that the legume portion in tortilla plays an important role in the content of this kind of nutraceutical compounds.

Hydrolysable polyphenols (HP) comprise hydrolysable tannins, phenolic acids, and hydroxycinnamic acids that are released from the food matrix by strong acid hydrolysis, no proanthocyanidins or flavonoids are detected in the hydrolysates of hydrolysable polyphenols analysis [39]. Saura-Calixto *et al.* [39] reported a HP content of 5.93 mg/g for legumes (35% chickpeas, 31% beans and 34% lentils), a value lower than that assessed here for black bean, this difference can be due to that different cultivar was studied. HP content of QPM tortilla was higher that those reported in yellow maize (4.47 mg/g) [38], the maize variety can be responsible of this difference, the value determined here is also higher that those reported in cereals (rice and wheat products) (4.72 mg/g) [39]. No difference in HP content was found between tortilla and black bean tortilla. Double cooking of bean could be responsible for this pattern, it has been reported that HP can be degraded by thermal action at high temperatures [43].

In general, total phenolic content (extractable and non-extractable polyphenols) in black bean tortilla was higher than the phenolic content of foods considered rich in polyphenols such as pepper (13.45 mg/g), broccoli (12.04 mg/g) and spinach (12.75 mg/g) [44].

### 2.5. Antioxidant Capacity

The antioxidant capacity (AC) of raw materials and tortillas is shown in Table 5. Extractable polyphenols of bean presented the highest AC level. Two black bean varieties showed an AC of 48.91 and 92.73 μmol Trolox eq/g, the bean variety is responsible of this difference [37]. The AC of EP of tortilla was similar to those reported for vegetables (6.7 μmol Trolox eq/g) [45]. An increase of 80.5% in antioxidant capacity of EP in black bean tortilla was found compared with tortilla. The AC of EP of black bean tortilla was similar to those reported for products with high AC such as red wine (12.14 μmol Trolox eq/ mL) [46].

The PA content in black bean was higher than EP content; however, the PA showed a lower antioxidant capacity, which could be due to differences in the chemical structures. The PA can be esterified with glucoside units in position C3 [47]. It has been observed that glycosylated flavonoids have minor antioxidant capacity [48]. Cooked black bean showed an antioxidant capacity of proanthocyanidins of 13.2 μmol Trolox eq/g [12], a value higher than that assessed here, the variety of the bean and the method of determination can be responsible of this difference. The AC of PA was not detected in tortilla. A similar result was reported by Pérez-Jiménez and Saura-Calixto [42] for cereals. In black bean tortilla, the AC of PA was detected, this is due to a dilution effect when the bean was mixed with maize, the value was similar that those reported for asparagus (3.92 μmol Trolox eq/g) and swiss chard stalk (3.53 μmol Trolox eq/g), the consumption of vegetables has been inversely associated with mortality from degenerative diseases [46].

No difference in AC of HP was found in tortilla and black bean tortilla. This pattern could be because HP in bean was degraded by thermal action in the second cooking [43].

In general, AC of total phenolic content (extractable and non-extractable polyphenols) in black bean tortilla was 17.61 μmol Trolox eq/g; if tortilla consumption is 328 g/day [3], the AC provided by black bean tortilla is equivalent to 3.56 mmol Trolox eq/day. Green tea is considered an important source of AC, and the AC of green tea is 6.01 mmol Trolox eq/L [46]. To reduce the risk to develop hypertension consumption of 120 mL/day of green tea (equivalent to 0.72 mmol Trolox eq/day) is suggested [49]. Consumption of 10 cups/day (150 mL/cup, 9.02 mmol Trolox eq/day) is linked with a decreased relative risk of death from cardiovascular disease [50]. It has been reported that polyphenols have anticancer [51] and antiproliferative [52] and antimutagenic activity [53].

## 3. Experimental Section

### 3.1. Sample Preparation

Black common bean seeds, cv. Negro 8025, were harvested in 2008 at the Bajio Experimental Station of the National Research Institute for Forestry, Agriculture and Livestock (INIFAP), located in Celaya, Guanajuato, Mexico. Seeds of cv. Ancho QPM maize were harvested in 2009 at the INIFAP, located in Iguala, Guerrero, Mexico. After dry bean Negro 8025 were cooked in water with NaCl (10 g/Kg bean) for 85 min at 97 °C, the grains and the cooking broth were lyophilized and then ground using a commercial grinder (Mapisa Internacional S.A. de C.V., Mexico, D.F.) to pass a US No. 50 sieve to produce the black bean flour. QPM maize grains were cooked in water (1:3, w/v) for 30 min at 97 °C at alkaline pH using lime (10 g/Kg grain), followed by cooling and soaking for 12 h. After soaking, QPM grains were washed three times with water, and grounded to make “masa” (QPM “masa”). Tortillas without cooked dry bean (QPM Tortilla) were produced with QPM “masa”. QPM “masa” was blended with black bean flour to produce a 70:30 ratio (w/w, dry matter) followed by addition of water to form “masa” with the consistency to made tortillas (QPM-black bean tortilla). The relationship masa:black bean was obtained of previous studies [13–15]. “Masa” was molded by pressure and extruded in commercial tortilladora MOT-G model (Tortilladoras González, Naucalpan, México) into thin circles to obtain 2 mm thick tortillas. Tortillas were cooked on a hot griddle for 1 min per side at an approximate temperature of 250 ± 5 °C. QPM “masa”, QPM tortilla and QPM-black bean tortilla were lyophilized and ground using a commercial grinder Universal model (Mapisa Internacional S.A. de C.V., Mexico, D.F.) to pass a US No. 50 sieve. The samples were stored at 10 °C until analysis.

### 3.2. Chemical Analysis

Ash, protein (N × 6.25) and fat were assessed according to AACC methods 08–01, 46–13 and 30–25, respectively [54,55]. Total dietary fiber was determined following the AOAC method 985.29 [55]. All analyses were performed in triplicate.

### 3.3. Total Starch

Total starch (TS) was determined by the method of Goñi *et al.* [34] with minor modifications; in brief, 50 mg of sample were dispersed in 2M KOH (60 min) to disperse all starch fractions, then samples were incubated with amyloglucosidase (No. 10 102857 001, Roche Diagnostics GmbH, Mannheim, Germany) at 60 °C and pH 4.75 for 90 min; glucose was determined using the glucose oxidase assay GOD-POD. TS were calculated as released glucose (mg) × 0.9.

### 3.4. Resistant Starch

Resistant starch (RS), including RS1 and RS2 fractions, was measured by Goñi *et al.* [56] in brief, protein and digestible starch were removal with pepsin (P-7000, Sigma Chemical Co., St. Louis, MO, USA) incubation (40 °C, pH 1.5, 1 h) and α-amylase (A-3176, Sigma Chemical Co.) incubation (37 °C, pH 6.9, 16 h). The residue was treated with 2 M KOH (30 min) and then incubated with amyloglucosidase (No. 10 102857 001, Roche Diagnostics GmbH) at 60 °C and pH 4.75 for 45 min. Glucose was determined using glucose oxidase/peroxidase assay (GPSL-0507, Elitech Clinical Systems). RS was calculated as glucose (mg) × 0.9. Digestible starch was calculated by difference between TS and RS.

### 3.5. *In Vitro* Kinetic of Starch Digestion

The *in vitro* rate of hydrolysis was measured using hog pancreatic α-amylase according to Holm *et al.* [57] with minor modifications. A 50 mL of phosphate buffer (pH 6.9) were added to a portion of each sample containing 500 mg of available starch. Samples were incubated a 37 °C in a shaking water bath. In the first 5 min before the addition of enzyme aliquots of 0.2 mL of each sample were taken to mark as time zero. After an interval of 1 min, 1 mL of a solution containing 40 mg of porcine pancreatic α-amylase (A-3176, Sigma Chemical Co.) in 1 mL of phosphate buffer was added to each sample. Samples (0.2 mL) were withdrawn after 15 min and every 15 min for 90 min. These samples were added to tubes than containing 0.8 mL distilled water and 1 mL of 3,5 dinitrosalicylic acid (DNS). Samples were incubated at 100 °C in water bath for 10 min. Then 15 mL of distilled water was added to each tube and mixed well. The reducing sugars released were measured at 530 nm in parallel with a standard curve of maltose. The rate of hydrolysis was expressed as the percentage of starch hydrolyzed with respect to dry matter at different times.

The predicted glycemic index (pGI) was calculated from percentage of starch hydrolyzed at 90 min (H_90_) values using the formula proposed by Goñi *et al.* [34]: pGI = 39.21 + 0.803 (H_90_) (*r* = 0.909, *p* ≤ 0.05).

### 3.6. Determination of Polyphenols Content

#### 3.6.1. Extractable Polyphenols

Samples (0.5 g) were extracted by constant shaking at room temperature with methanol:water acidified with HCl (50:50 v/v, pH 2, 50 mL/g sample, 60 min) and acetone:water (70:30 v/v, 50 mL/g sample, 60 min). After each extraction step, samples were centrifuged (15 min, 25 °C, 3000 g) and supernatants were combined and used to determine extractable polyphenols content by the Folin-Ciocalteau procedure [58]. The results were expressed as gallic acid equivalents.

#### 3.6.2. Non Extractable Polyphenols

##### 3.6.2.1. Proanthocyanidins

Residues from the methanol/acetone/water extraction were treated with 5 mL/L HCl in butanol for 3 h at 100 °C for proanthocyanidins determination [59]. Proanthocyanidins were calculated from the absorbance at 550 nm of the anthocyanidin solutions. Proanthocyanidins from Mediterranean carob pod (*Ceratonia siliqua* L.) supplied by Nestlé S.A. were treated under the same conditions to obtain standard curves.

##### 3.6.2.2. Hydrolysable Polyphenols

Hydrolysable polyphenols were determined by a methanol/H_2_SO_4_ 90:10 (v/v) hydrolysis at 85 °C for 20 h on the residues of the methanol/acetone/water extraction [60]. After centrifugation (15 min, 25 °C, 3000 g) supernatants were used to determine hydrolysable polyphenols by the Folin-Ciocalteau procedure [58]. The results were expressed as gallic acid equivalents.

### 3.7. Free Radical-Scavenging Assay (ABTS)

The antioxidant capacity of extractable polyphenols, proanthocyanidins, and hydrolysable polyphenols extracted from samples were estimated in terms of radical-scavenging activity following the procedure described elsewhere [61] with some modification [62]. Briefly, ABTS [2,2′-azinobis-(3-ethyl-benzothiazoline-6-sulfonic acid)] radical cation (ABTS^+•^) was produced by reacting 7 mM ABTS stock solution with 2.45 mM potassium persulphate in the dark at room temperature for 12–16 h before use. The ABTS^+•^ solution was diluted with methanol to an absorbance of 0.70 ± 0.02 at 730 nm. After addition of 0.1 mL of extract to 3.9 mL of diluted ABTS^+•^ solution, absorbance readings were taken every 20 s using a UV-1800 UV-vis spectrophotometer (Shimadzu Europe GmbH, Duisburg, Germany). The reaction was monitored for 6 min. Inhibition of absorbance versus time was plotted, and the area below the curve (0–6 min) was calculated. Results were expressed as μmol of Trolox equivalents per g of dry matter.

### 3.8. Statistical Analysis

Results are presented as mean ± SEM (standard error of mean) of three separate determinations. A commercial software program (SigmaPlot for Windows version 11.0, San Jose, CA, USA) was used to evaluate, by one-way analysis of variance, significant differences in the means of measured parameters. Statistically significant differences (*p <* 0.05) amongst means were evaluated using the Tukey multiple comparison procedure.

## 4. Conclusions

The addition of bean to tortilla made with quality protein maize improved the protein, ash and dietary fiber content. The starch digestibility rate of tortilla with bean decreased and, in consequence, produced lower predicted glycemic index. QPM Tortilla with black dry bean showed higher antioxidant capacity than QPM tortilla. The tortilla with bean can be an alternative for people with particular nutritional or metabolic requirements.

## Figures and Tables

**Table 1:** Chemical composition of black bean flour, quality protein maize (QPM) masa, QPM tortilla and QPM-black bean tortilla (g/100 g).

Sample	Protein *	Fat	Ash	TDF
Black bean flour	21.45 ± 0.36 a	1.24 ± 0.03 a	4.66 ± 0.02 a	36.24 ± 0.70 a
QPM masa	7.89 ± 0.11 b	5.03 ± 0.22 b	1.61 ± 0.02 b	8.55 ± 0.41 b
QPM tortilla	8.78 ± 0.06 b	4.37 ± 0.23 bc	1.67 ± 0.00 b	9.36 ± 0.27 b
QPM-black bean tortilla	12.04 ± 0.43 c	3.89 ± 0.25 c	2.58 ± 0.01 c	14.72 ± 0.30 c

Values are mean ± SEM, n = 3, dry matter. Means in the same column with different letter are significantly different (p < 0.05). TDF: Total dietary fiber.

* N × 6.25.

**Table 2:** Total starch, digestible starch and resistant starch in black bean flour, quality protein maize (QPM) masa, QPM tortilla and QPM-black bean tortilla (g/100 g).

Sample	Total starch	Digestible starch*	Resistant starch
Black bean flour	44.36 ± 0.52 a	37.92 ± 0.55 a	6.44 ± 0.07 a
QPM masa	77.68 ± 0.20 b	74.50 ± 0.20 b	3.17 ± 0.01 b
QPM tortilla	76.69 ± 0.82 b	71.65 ± 0.80 c	5.04 ± 0.05 c
QPM-black bean tortilla	66.75 ± 0.47 c	60.45 ± 0.48 d	6.30 ± 0.08 a

Values are mean ± SEM, n = 3, dry matter. Means in the same column with different letter are significantly different (p < 0.05).

* Values calculated as the difference between total starch and resistant starch.

**Table 3:** Predicted glycemic index (pGI) of black bean flour, quality protein maize (QPM) tortilla and QPM-bVlack bean tortilla.

Sample	Hydrolysis at 90 min (%)	pGI *
Black bean flour	10.77 ± 0.35 a	47.92 ± 0.27 a
QPM tortilla	60.72 ± 0.27 b	87.97 ± 0.22 b
QPM-black bean tortilla	50.50 ± 0.23 c	79.76 ± 0.18 c

Values are mean ± SEM, n = 3, dry matter. Means in the same column with different letter are significantly different (p < 0.05).

* Predicted glycemic index (pGI) = 39.21 + 0.803(H_90_) [34].

**Table 4:** Polyphenols content of black bean flour, quality protein maize (QPM) tortilla and QPM-black bean tortilla.

Sample	Polyphenols content (mg/g)		

	EP	PA	HP
Black bean flour	5.55 ± 0.08a	22.94 ± 2.12a	12.13 ± 0.23a
QPM tortilla	0.96 ± 0.05b	N.d.	5.70 ± 0.13b
QPM-black bean tortilla	1.99 ± 0.08c	8.68 ± 1.28b	6.17 ± 0.18b

Values are mean ± SEM, n = 3, dry matter. Means in the same column with different letter are significantly different (p < 0.05). EP: Extractable polyphenols; PA: Proanthocyanidins; HP: Hydrolysable polyphenols; N.d.: Not detected.

**Table 5:** Antioxidant capacity of black bean flour, quality protein maize (QPM) tortilla and QPM-black bean tortilla.

Sample	Antioxidant capacity (ABTS method) (μmol Trolox eq/g)		

	EP	PA	HP
Black bean flour	57.58 ± 0.18 a	11.27 ± 0.65 a	14.54 ± 0.24 a
QPM tortilla	6.95 ± 0.49 b	N.d.	0.87 ± 0.46 b
QPM-black bean tortilla	12.55 ± 0.42 c	3.62 ± 0.23 b	1.44 ± 0.35 b

Values are mean ± SEM, n = 3, dry matter. Means in the same column with different letter are significantly different (p < 0.05). EP: Extractable polyphenols; PA: Proanthocyanidins; HP: Hydrolysable polyphenols; N.d.: Not detected.
